# Anticipation of pain enhances the nociceptive transmission and functional connectivity within pain network in rats

**DOI:** 10.1186/1744-8069-4-34

**Published:** 2008-08-26

**Authors:** Jin-Yan Wang, Han-Ti Zhang, Jing-Yu Chang, Donald J Woodward, Luiz A Baccalá, Fei Luo

**Affiliations:** 1 Key Laboratory of Mental Health, Institute of Psychology, Chinese Academy of Sciences, Beijing, PR China; 2 Neuroscience Research Institute, Peking University, Beijing, PR China; 3 Neuroscience Research Institute of North Carolina, Winston-Salem, NC, USA; 4 Telecommunications and Control Engineering, Escola Politecnica, University of Sao Paulo, Brazil

## Abstract

**Background:**

Expectation is a very potent pain modulator in both humans and animals. There is evidence that pain transmission neurons are modulated by expectation preceding painful stimuli. Nonetheless, few studies have examined the influence of pain expectation on the pain-related neuronal activity and the functional connectivity within the central nociceptive network.

**Results:**

This study used a tone-laser conditioning paradigm to establish the pain expectation in rats, and simultaneously recorded the anterior cingulate cortex (ACC), the medial dorsal thalamus (MD), and the primary somatosensory cortex (SI) to investigate the effect of pain expectation on laser-induced neuronal responses. Cross-correlation and partial directed coherence analysis were used to determine the functional interactions within and between the recorded areas during nociceptive transmission. The results showed that under anticipation condition, the neuronal activity to the auditory cue was significantly increased in the ACC area, whereas those to actual noxious stimuli were enhanced in all the recorded areas. Furthermore, neuronal correlations within and between these areas were significantly increased under conditions of expectation compared to those under non-expectation conditions, indicating an enhanced synchronization of neural activity within the pain network. In addition, information flow from the medial (ACC and MD) to the lateral (SI cortex) pain pathway increased, suggesting that the emotion-related neural circuits may modulate the neuronal activity in the somatosensory pathway during nociceptive transmission.

**Conclusion:**

These results demonstrate that the nociceptive processing in both medial and lateral pain systems is modulated by the expectation of pain.

## Background

Pain is a personal and subjective experience. The psychological factors therefore play important roles in shaping pain perception. One of these factors is expectation. In clinical situations, when pain is anticipated, patients often report the worsening of pain [1-3]. Conversely, expectation of pain relief is considered to be effective means for producing placebo analgesia [4-6]. Recently, Keltner *et al. *demonstrated that the level of expected pain intensity significantly altered perceived pain when the comparison was made between two noxious thermal stimuli of almost equal intensity [7].

Interests in pain anticipation-related brain activity has increased in recent years. EEG recording revealed significantly enhanced signals during anticipation of painful stimuli or priming with pain-related adjectives [8,9]. Functional imaging studies suggested that expectation of pain could cause alteration in both pain perception and forebrain pain transmission [10-12], even amplify brain responses to nonpainful somatosensory stimulation [13]. More interestingly, the pain anticipation-related areas are largely overlapped with pain-related areas, such as the primary somatosensory cortex (SI), anterior cingulate cortex (ACC), periaqueductal grey (PAG), insular cortex (IC), prefrontal cortex (PFC), and cerebellum [10-12,14,15].

Although expectation of pain has been extensively studied in humans using neuroimaging techniques, neural mechanisms underlying the modulating effect of expectation are far from clear. Functional imaging studies are able to detect expectation-related signal changes, but can not directly measure neuronal spike activity. In fact, the correlation between imaging signals and action potential firing of neurons is still unclear [16]. Furthermore, activation of brain sites revealed by imaging studies can not resolve issues as to how the information is transferred among the brain regions. The issues can only be addressed in animal experiments [17].

We reported previously that the medial and lateral pain pathways were activated in parallel manner by cutaneous noxious radiant heat in awake rats, using a multiple-channel single-unit recording method [18]. In that study, we incidentally observed anticipatory responses in the medial pain system, including the ACC and medial dorsal thalamus (MD). To further explore this issue, we investigated the effect of pain anticipation on nociceptive behavior and neural activity in awake rats. To establish anticipation in rats, we employed a Pavlovian conditioning paradigm, in which a neutral conditioned stimulus (tone) was paired with a noxious unconditioned stimulus (laser). The establishment of pain expectation was determined by the acquisition of conditioned responses (tone-induced avoidance). The aim of this study was to determine whether and how pain expectation could alter nociceptive processes and functional connectivity in the nociceptive neural networks.

## Results

### Behavioral response

**Figure 1:**
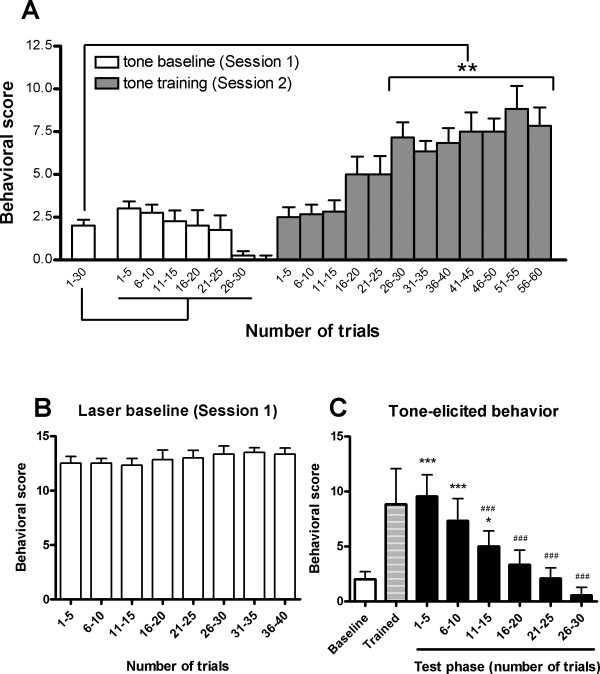
**Tone or laser-elicited behavior in the first two sessions**. (A) The learning effect demonstrated by tone alone-elicited behavior. The behavioral score was accumulated every 5 successive trials. One-way ANOVA followed by the *Dunnett *test for multiple comparisons were used to compare the difference of behavioral scores between Session 2 and 1 (* *p *< 0.05). As can be seen, rats learned to escape immediately after the tone after about 25 tone-laser pairing trials. (B) Laser-induced nociceptive behavior in the first session. (C) The acquisition and extinction of the conditioned response demonstrated by tone alone-elicited behavior. *, *** *p *< 0.05, *p *< 0.001, respectively, compared with "Baseline"; ###, *p *< 0.001, compared with "Trained", one-way ANOVA followed by the Newman-Keuls Multiple Comparison Test. "Baseline" and "Trained" are the averaged behavioral scores in the first and second sessions (trials 1–30 in Session 1 and trials 51–55 in Session 2, see above Fig. 1A), respectively.

Rats responded to auditory stimuli with high level exploratory activity in the early stage of Session 1 as evidenced by rearing, head movement, and short-lasting freezing. After repeated tone presentation, these exploring behaviors substantially subsided, as Fig. 1A (Session 1) illustrated. By contrast, noxious laser stimulation caused marked escaping responses such as foot jumping, lifting and licking. Such nociceptive behaviors always occurred throughout the first session (Fig. 1B).

In the second session, rats received tone-laser conditioning training. As can be seen in Fig. 1A (Session 2), rats gradually learned to escape after tone but prior to the delivery of noxious stimulation. Following 25 tone-laser pairing trials, the auditory cue became a reliable predictor for the forthcoming painful stimuli. In the testing phase, it was observed that the tone alone was able to elicited escaping behavior (Fig. 1C), indicating the acquisition of the conditioned response. Interestingly, at the same time when rats are sensitive to the warning signal, the nociceptive behavior induced by actual pain stimulation was significantly reduced compared to Session 1 (9.18 ± 1.05 *vs. *12.92 ± 0.16, *p *< 0.05).

### Laser- and tone-induced neuronal activity

**Figure 2:**
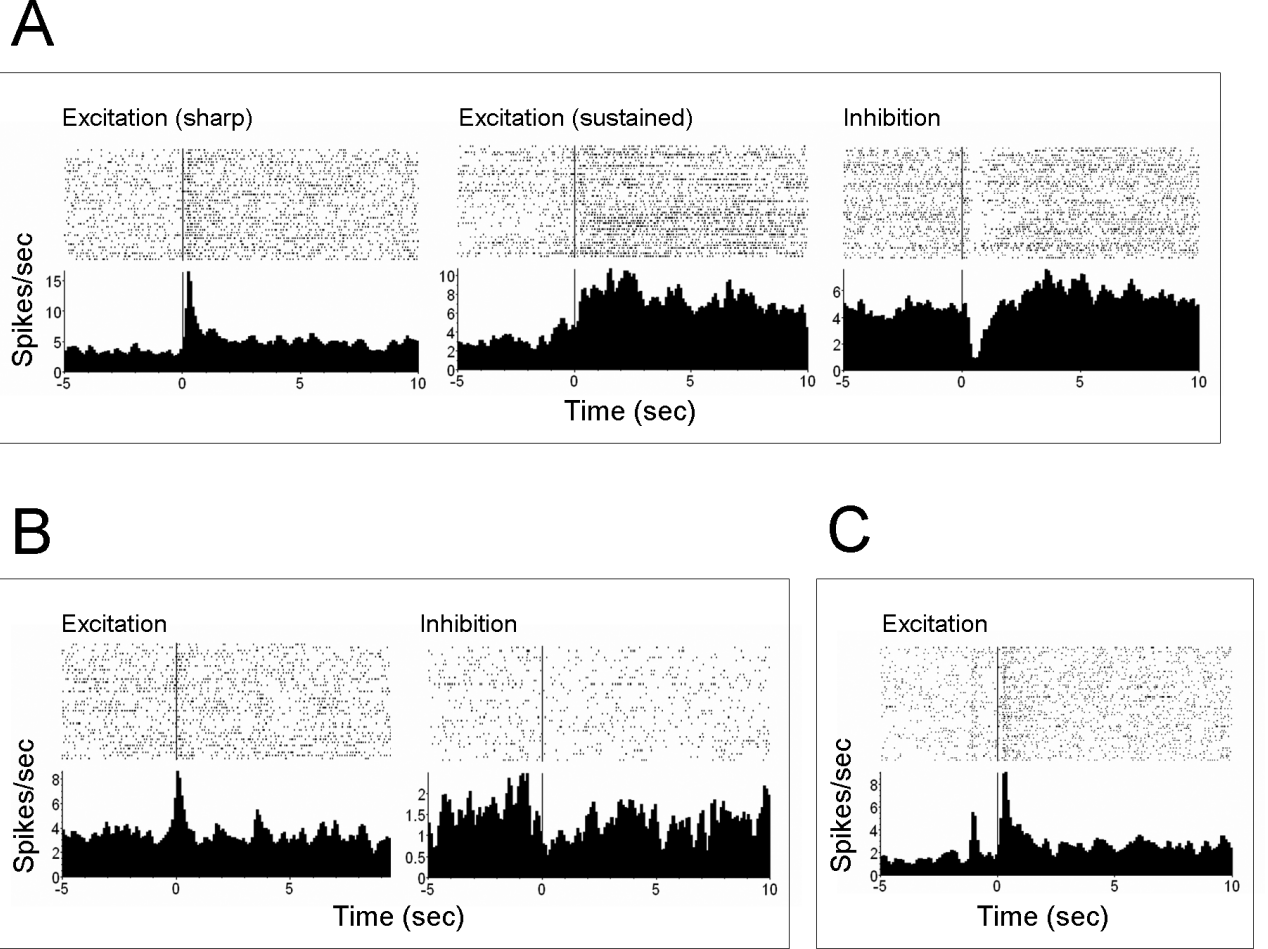
**The typical neuronal response elicited by simple laser (A), simple tone (B), and paired tone and laser (C)**. PSTHs illustrated the average firing rate of a neuron around a stimulus. Time = 0 on the x-axis corresponded to the time of noxious (A, C) or tone (B) stimulus onset.

A total of 216 – 224 single units were simultaneously recorded (72 – 73 from the ACC, 61 – 64 MD, and 83 – 87 SI, varied between sessions due to neuron drifting). Noxious laser induced predominantly excitatory responses, displayed in sharp or sustained manner, as shown in Fig. 2A. The neurons exhibiting excitatory responses accounted for 32%, 51%, and 60% in ACC, MD thalamus, and SI cortex, respectively. Inhibitory neuronal responses were occasionally encountered and less than 5%. Auditory stimuli also elicited discharge of a small proportion of neuron within the recorded areas, with 7% in ACC, 16% in MD, and 11% in SI, as shown in Fig. 2B. Fig. 2C illustrated the typical neuronal response produced by tone-laser pairing.

**Figure 3:**
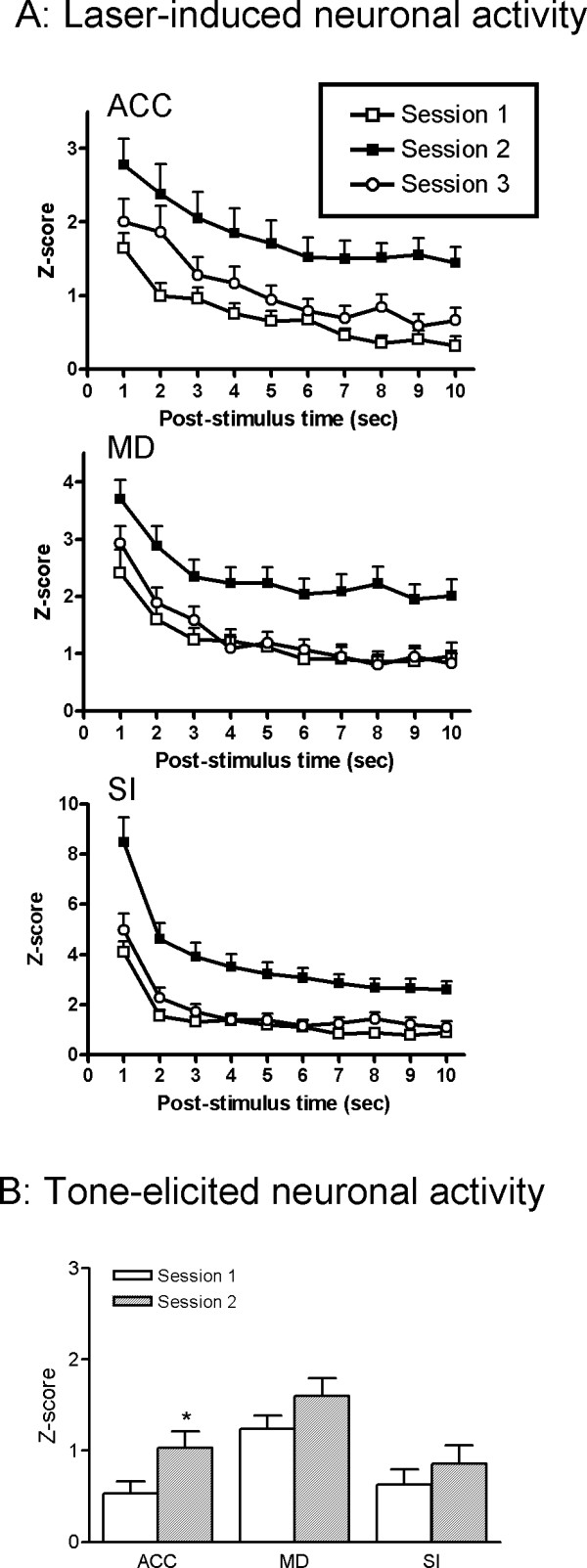
**Laser (A) and tone-induced (B) responses in each session**. The magnitude of neuronal discharge was assessed by *Z*-scores. The laser-induced response was presented as a time course post laser stimulus and averaged every 1 sec. The tone-elicited response was calculated 0 – 1 s following the onset of the tone. *, # *p *< 0.05 indicate significantly different from Session 1 and Session 3, respectively.

We compared the laser-induced neuronal responses during post-stimulus time across the three sessions. As shown in Fig. 3A, a significant difference was detected across sessions (ACC, *F*(2,2130) = 59.74, *p *< 0.0001; MD, *F*(2,1850) = 63.57, *p *< 0.0001; SI, *F*(2,2520) = 109.2, *p *< 0.0001). A post hoc *Bonferroni *test for multiple comparisons showed that the pain-related responses in Session 2 were significantly higher than in Session 1 (*p *< 0.05) for all the recorded areas, suggesting that anticipation of pain may enhance the nociceptive transmission in the brain. No significant difference was found between Sessions 1 and 3, indicating that no sensitization or tolerance was developed throughout Session 1–3. Tone-related responses were relatively weak with respect to the laser-induced responses. As illustrated in Fig. 3B, comparing with tone presentation alone, paring the tone cue with nociceptive stimulation significantly increased tone-related neural activity in the ACC but not in the MD and SI. These results suggest that the ACC may play a significant role in neural processing involved in pain anticipation.

### Functional connectivity within and between the recorded areas during noxious stimulation

**Figure 4:**
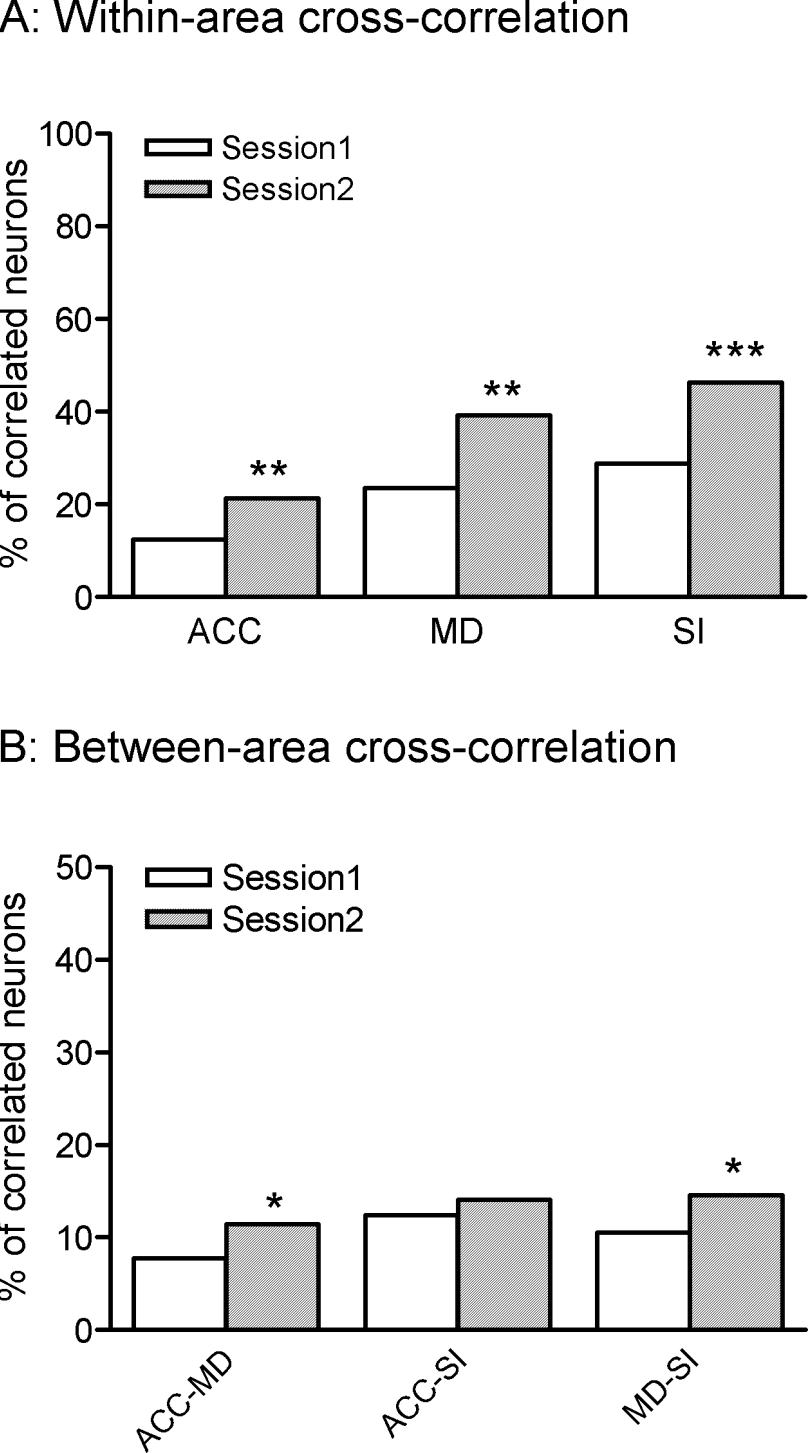
**The percent of significantly correlated neuronal pairs within (A) and between (B) recorded brain areas during noxious stimulation**. * *p *< 0.05 indicates significantly different from Session 1.

Correlations between the neurons within the same region were observed more often than those between different regions. For the within-area cross-correlations, *Chi*-square tests showed that the correlated activity in Session 2 was significantly higher than that in Session 1 (*p *< 0.05) for all the recorded areas (Fig. 4A). For the between-area cross-correlations, the significantly enhanced correlated activity was observed between the MD and the other two regions (ACC and SI) in session 2 in comparison with session 1 (Fig. 4B).

### Information flow between the recorded areas during noxious stimulation

**Figure 5:**
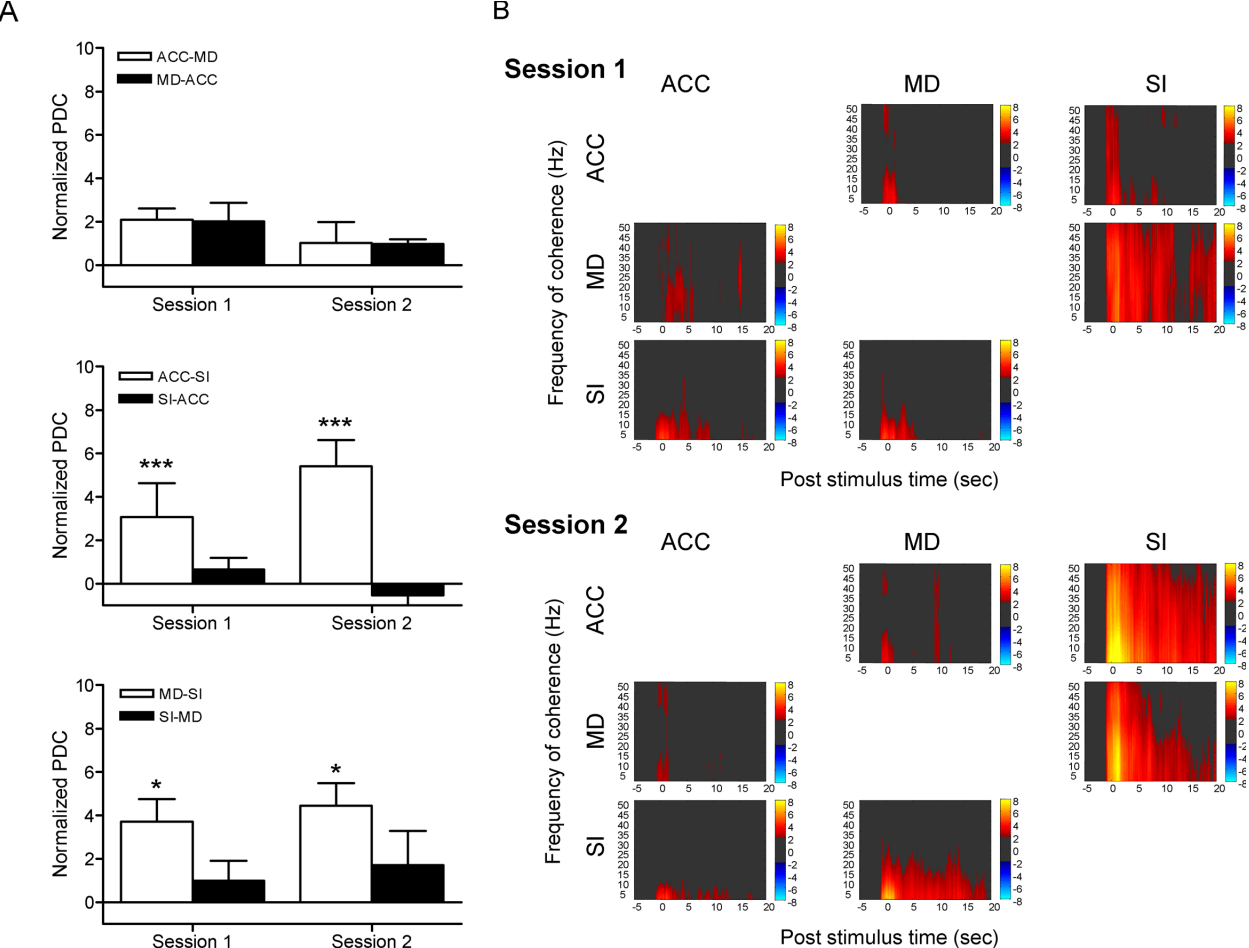
**Result of partial directed coherence analysis during noxious stimulation**. (A) Two-way ANOVA showed that the information flow from ACC to SI, and MD to SI was significantly larger than that in the opposite direction in both Session 1 and 2. 'ACC-MD' indicates directed coherence from ACC to MD, and the same as the other regional pairs. Values are normalized to the pre-stimulation baseline level. * *p *< 0.05, *** *p *< 0.001, compared with PDC in the opposite direction. (B) An example of the amount of partial directed coherence observed between recorded areas. These PDC values were normalized to z-scores relative to the mean and variance of baseline (pre-stimulation) PDC. The normalized PDCs exceeding 95% confident interval of the baseline were displayed in pseudo colour. Warm and cool colours indicate the increase and decrease in PDC, respectively. The direction of information flow are from the column area to the row area.

PDC analysis was used to determine the direction of information flow from one region to others under different experimental conditions. A two-way ANOVA was performed to measure the difference in the normalized PDC between the Session 1 and 2. There was no significant Session × Direction interaction for all the regional pairs. However, significant effect was found in directions for ACC-SI (*F*(1,31) = 13.86, *p *= 0.0008) and MD-SI (*F*(1,30) = 5.184, *p *= 0.0301), which indicated that the information flow from the ACC to SI, and the MD to SI was significantly larger than that in the opposite direction in both sessions (Fig. 5A and 5B).

## Discussion

In the present study, simultaneous single unit recording was performed in the ACC, MD and SI to study the neural mechanism underlying the effect of pain expectation using tone-laser conditioning model in rats. There were three main findings. First, under anticipation condition, neuronal responses to the auditory cue were significantly increased only in the ACC whereas those to nociceptive stimuli were enhanced in all the recorded areas. Second, expectation of impending pain enhanced correlated neural activity within and between recording areas following noxious stimuli and third, there were larger amounts of information flow from the medial (ACC and MD) to the lateral (SI cortex) pathway during pain processing.

Neuroimaging studies have identified anticipation-related activation in many cortical areas including the SI, ACC, IC, and the PFC [10-12,14,15]. In the present study, we found significant increase in the neuronal response in the ACC but not in the SI during pain expectation. There are two possible explanations for the inconsistency between our and others' results. First, the animal model used in the present study is the type of 'certain' expectation, in which the neutral conditioned stimulus (tone) reliably predict the noxious unconditioned stimulus (laser). In contrast, most imaging studies on human expectation employed an uncertain paradigm. Certain and uncertain expectations have been demonstrated to be mediated by different neural pathways; the former is associated with activity in the ACC [10,15,19-21], whereas the latter involves changes in the SI [11,14,22]. Thus, our results provide evidence that the ACC, rather than the SI, is a structure critically involved in the neural process underlying certain expectation of pain. Another possible explanation is derived from the attention-related focusing mechanism. Previous studies in rats have found that the ACC is involved in tasks required visual or audio attention and preferentially activated during presentation of the conditional stimulus [23-27]. The increased activity in the ACC observed in the present study could also be due to the conditioning experiment employed.

As previously described, expectation of an aversive event (painful stimulation) can modify subsequent behavior (pain reactivity). Conditioned expectation (certain expectation) is associated with the emotional state of fear, which produces hypoalgesia [28-30]. In contrast, unconditioned expectation (uncertain expectation) is related to anxiety, which has the opposite effect on pain, i.e., hyperalgesia [31-33]. In our study, we found that the nociceptive behaviors (paw lifting and licking) induced by actual pain stimulation were reduced during conditioning, which is consistent with prior studies that the conditioned fear leads to decreased behavioral reactivity [33]. Unexpectedly, the analysis of neuronal activity suggested that the laser-elicited responses in all recorded areas were enhanced under the expectation conditions. This seemed in contradiction with the behavioral findings that pain was decreased. It should be noted that noxious stimulation-elicited fear itself is a negative emotion. The emotional component of pain has been known to involve pathways through the medial thalamus to the ACC [34,35]. In addition, emotional states are found to be closely related to attentional states [12]. There is evidence in humans and animals for the involvement of the ACC as well as the primary somatosensory cortices in the attentional modulation of pain [36-38]. Thus, the neuronal responses in the recorded areas may reflect a mixed effect exerted by expectation. On the other hand, the increased neuronal activity and cross-correlations in ACC and MD during noxious stimulation may represent an endogenous antinociceptive activation instead of signalling nociceptive information. Early studies indicate that both the medial thalamus and ACC are involved in the activation of descending pain suppression mechanisms. Projections from the midline thalamic nuclei and ACC to the PAG have been described [39,40]. A high density of opioid receptors and activation induced by fentanyl within ACC support the participation of it in the down-regulation of pain perception [41]. Therefore, the inconsistence between the behavioral findings and neural activity change suggest a more complex role of medial system in pain processing, including both mediation and suppression.

Converging evidence indicates that pain is a multi-dimensional experience that involves distributed brain regions comprising lateral and medial systems [42-45]. The medial pain system consists of the ACC and the medial thalamic nuclei and is believed to process the emotional-motivational component of pain [19,35]. The functional relationship and anatomical connection between the ACC and the medial thalamus have been demonstrated by numerous studies [46-50]. The present study simultaneously recorded neurons in these areas, and found a significant increase in the number of correlated neuronal pairs within the same and between different brain areas under anticipation conditions, compared to those under non-anticipation conditions. An increased synchronized activity observed in the present study suggests temporal coding may play a significant role in processing pain perception under the conditioning state. Together with previous discussion, these results indicated an enhanced network processing in the pain neuromatrix under the expectation of pain. Further studies will be required to elucidate the implication of this finding.

Another interesting finding of this study is larger amount of information flow from the medial (ACC and MD) to the lateral (SI cortex) pathway as compared to those in the opposite direction. PDC analysis reveals causality of coherent neural activity of two regions. In this view, our result indicates that the emotion-related neural circuits may modulate the neuronal activity in the somatosensory pathway during nociceptive transmission. Our previous study on tonic pain has demonstrated an increase in the information flow from the medial to the lateral pain pathway during the first hour after formalin injection [51]. Although little available evidence supports direct linkage between the medial and the lateral pain systems [34], our prior and current results both suggest the medial system may modulate lateral system during nociceptive processing, Based on the fact that the medial system is composed of the medial and intralaminar thalamic nuclei and limbic cortical areas which have descending projections to nociception regulating centres such as PAG, it is possible that the medial system modulates somatosensory nociceptive transmission through the brainstem structures that control both spinal and trigeminal dorsal horn pain transmission neurons. Thus, clarifying the anatomical and functional interaction between the parallel systems can provide deeper insight into the neural mechanism of expectation related pain modulation and may help us to improve the treatment of clinical pain.

## Conclusion

The present study demonstrated that anticipation of pain enhanced the neuronal discharges and correlated neural activity within and between brain regions in the medial and lateral pain pathways induced by the following noxious stimuli, indicating that the nociceptive processing in both medial and lateral pain systems is modulated by the expectation of pain.

## Methods

### Animals

All experiments were performed on nine male Sprague-Dawley rats (250–300 g) individually housed in a room maintained with a 12-h light/dark cycle. Food and water were available *ad libitum*. The experimental protocols were approved by the Animal Care and Use Committee at the Chinese Academy of Sciences and in accordance with the IASP guidelines for animal study. Every effort was made to minimize both animal suffering and the number of animals used.

### Surgery for microelectrode implantation

After a 7-day period of habituation, animals received the surgery of microelectrode implantation. As in the previously described procedure [18], rats were anesthetized with ketamine (100 mg/kg, i.p.) and xylazine (10 mg/kg i.m.) and mounted on a stereotaxic frame. Following the retraction of skin and soft tissue, small holes corresponding to the recording sites were drilled on the skull. Then arrays of eight stainless steel Teflon-coated microwires (50 μm diameter, Biographics, Inc. Winston-Salem, NC, USA) were implanted unilaterally into the target brain areas. The coordinates were as follows: 3.2 mm anterior (A) to bregma, 0.8 mm lateral (L) to midline, 2.5 mm ventral (V) from the skull surface for the ACC; 2.3 mm posterior (P), 0.8 L, 5.5 V for the MD; and 1.0 P, 2.0 L, 2.0 V for the SI cortex. The microarrays were fixed in place with dental cement. Animals were housed individually and allowed to recover from surgery for at least 7 days before being subjected to the experiment.

### Apparatus and laser stimulation

The experimental chamber was 44 × 22 × 44 cm in dimension and made of transparent acrylic plastics. The floor of the chamber contained an array of holes (diameter 6 mm) spaced 10 mm apart (centre to centre). A computer-controlled CO_2 _laser stimulator (Model DM-300, Changchun Institute of Optics, Fine Mechanics and Physics, Chinese Academy of Science) was used to deliver pain stimuli. The laser radiation was 10.6 μm in wavelength and 2.5 mm spot diameter. The output power and duration of the stimulation were set at 8 W and 20 ms, which was designed to activate the primary nociceptive afferents without damaging the skin or the subcutaneous tissue [52]. The CO_2 _laser was equipped with a He-Ne aiming beam, which was visualized prior to firing the laser. The laser beam was emitted through the holes at the floor of the chamber to the plantar surface of rat hindpaw contralateral to the recording brain regions. A tone generator (Coulbourn Inc. USA) was mounted on the side wall of the chamber for audio stimuli (80 dB, 800 Hz, 100 ms). In tone-laser pairing trials, a laser pulse was delivered 900 msec after the tone. The onsets and durations of the tone and laser were controlled by a PC running program *Magnet *(Biographics, Inc.). A video camera was positioned in front of the chamber to record behavioral activity.

### Experimental protocol

**Figure 6:**
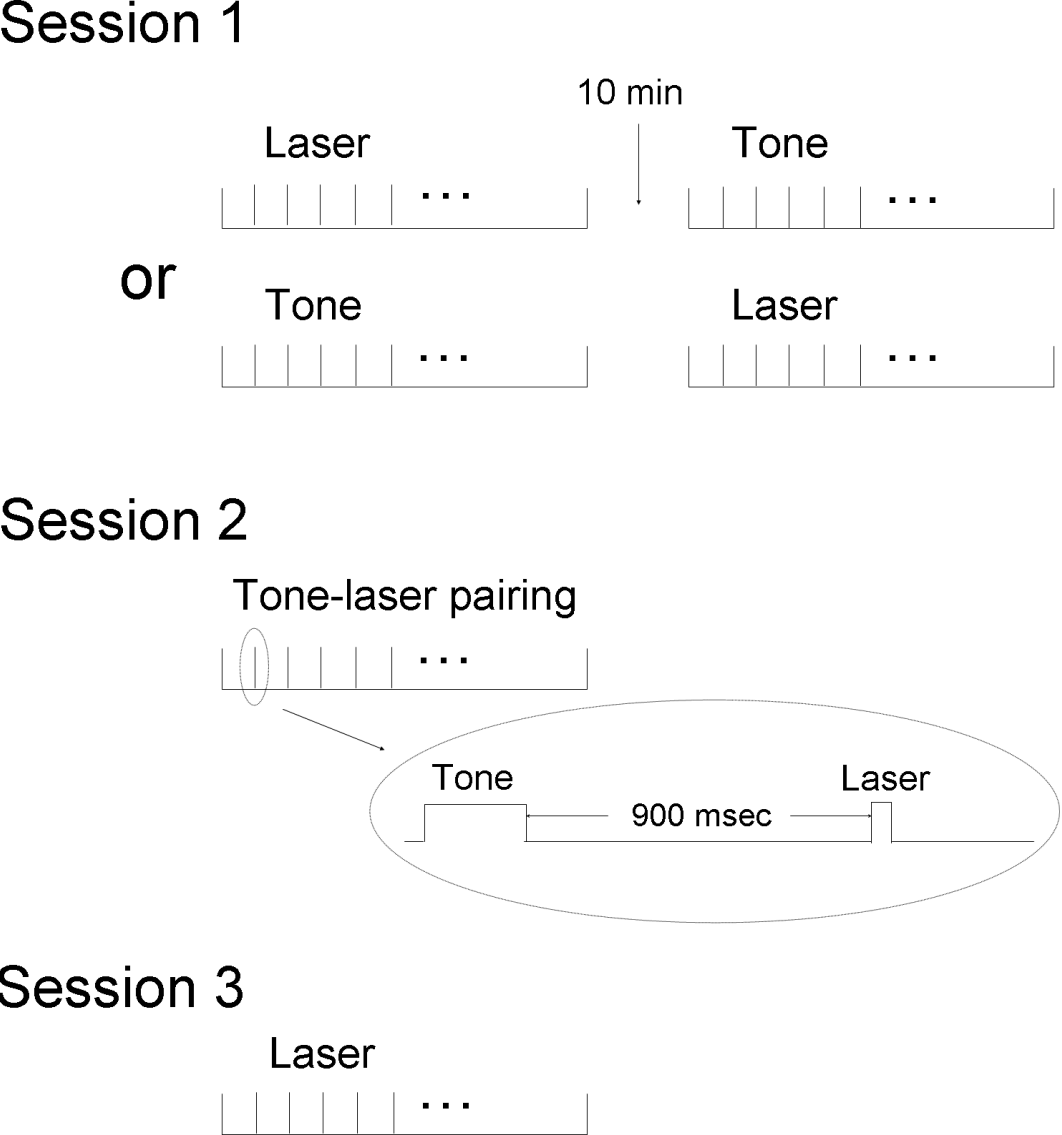
**Experimental procedure**. Session 1 contains two blocks of stimuli, one consisted of 40 laser stimuli and the other consisted of 30 tone stimuli. Session 2 involves 60 tone-laser pairing stimuli trials. Session 3 comprises another 40 trials of laser stimuli. The inter-trial interval was no less than 60 sec.

The experiments were conducted under normal lighting. In the first 3 days, rats were placed individually in the experimental chamber each day for 1 h. The experimental sessions started from day 4. All rats underwent 3 experimental sessions in 3 successive days (Fig. 6). The behavior of rats was continuously videotaped throughout the sessions for later analysis.

In Session 1, two blocks of stimuli were delivered. One consisted of 40 painful laser stimuli and the other consisted of 30 tone stimuli, with inter-trial interval of no less than 60 sec. The order of the stimuli was balanced across animals. This session serves as a normal control for the following two sessions.

In the second session, animals were subjected to 60 tone-laser pairing stimuli trials. This conditioning training formed a stable linkage between auditory cues and animals' nociceptive behaviors, i.e. the auditory cue will forecast the impending painful stimulus. This learning phase was followed by a testing phase (30 trials), in which the tone was administered without paired with laser, to determine the acquisition and extinction of the learned responses. The Session 2 was used to assess the effects of anticipation on neural activity and functional connectivity within central pain networks.

In Session 3, animals received another 40 trials of laser stimuli. This session tests the possibility of sensitization or tolerance for noxious stimuli.

### Unit recording

The simultaneous extracellular recording of the three selected brain areas was performed throughout the experimental sessions. Neural electric signals were obtained from the stainless steel microwires and passed from the headset assemblies to a preamplifier via two lightweight cables and a commutator. The commutator was free to turn as necessary, permitting unrestricted movement of the rat. The signals were band-pass filtered between 0.5 and 5 kHz (6 dB cutoff) before being sent to a spike-sorting device (Biographics, Inc.). Valid spikes were selected using amplitude and duration thresholds and recorded into a database file with PC-based software (*Magnet*, Biographics, Inc.). The identity of clearly sorted single neurons was verified by graphical capture of waveforms. Onset of tone and stimulation events was recorded into the data file with a resolution of 1 ms.

### Histology

Animals were sacrificed with overdosed pentobarbital at the end of experiment. Recording sites were marked by electrophoretically deposited iron (20 μA, 10 – 20 s) at the tips of selected wires. Animals were then perfused with 4% paraformaldehyde and the brains were post-fixed, frozen, and cut coronally into 40-μm sections. The iron deposits could be visualized as blue dots under light microscope. Data obtained from the microwires outside the target regions were not included in the analysis.

### Behavioral assessment and analysis

Behavioral responses to nociceptive stimuli were assessed by off line video analysis. According to the method of Fan *et al.*, the laser-induced nociceptive responses in rats can be classified as eight categories: head movement (Hm), body movement (Bm), foot jumping (Fj), foot elevation (Fe), foot movement (Fm), licking (Li), rearing (Re), and grooming (Gr) [52]. The frequency and duration of each response were then used to quantitatively evaluate nociceptive behaviors. Here we modified the method by focusing on five of the eight categories listed above, i.e., Hm, Bm, Fj, Fe, and Fm. A score of 0 was assigned if the rat stayed quietly; a score of 1 if the rat displayed Hm (exploring); a score of 2 if Bm or Fm (motivation of avoidance) was observed; a score of 3 if Fj or Fe (successful escape) occurred. The behavioral response was measured with cumulative scores every 5 successive trials. One-way ANOVA followed by the *Dunnett *test for multiple comparisons was used to compare behavior scores between sessions. The behavioral assessment was used to examine whether and when the tone-laser association was steadily formed.

### Data analysis

The neuronal firing rate was quantified for each neuron using peri-event time histograms (PSTHs). The bin size was 0.1 s for the computation of PSTHs. Bin counts for each trial were calculated using the analysis program NeuroExplorer (Plexon, Dallas, TX) and the results were exported to Matlab (The MathWorks, Inc.) in spreadsheet form. Neural responses to auditory or noxious stimulation were evaluated using a sliding window averaging technique, in which a 1-s time window was slid through the entire period of a trial at 0.1-s step. The bin counts of each window were compared with those of a preset 3-s control window 10 s before the stimulation event by Student's *t*-test. The differences were considered significant only when it reached a significance level of *p *< 0.005 in three consecutive steps, thus to achieve a global significance of *p *< 0.05. Units that significantly increased their activities after tone or laser stimuli were defined as excitatory; those that decreased their activities were considered inhibitory. To compare the neural responses between different sessions, the neuronal firing rates were transferred into Z scores using MatLab program: Z = (X-M)/S, where X is the actual firing rate obtained from PSTH, M and S are mean and standard deviation of the baseline discharging (-5 – -2 s), respectively.

To identify the functional interactions between the recorded areas, cross-correlation and partial directed coherence (PDC) analyses were performed. In the cross-correlation analysis, one neuron was selected as the reference neuron and all other neurons were defined as partner neurons. The peri-spike histogram of a partner neuron within -0.5~0.5 sec around the reference neuron were calculated with a 5-ms bin size and a 3-bin *Gaussian *smooth. The significance level of the cross-correlograms was defined by 95% confidence level in the *Nex *program. Data falling into the 10-s period after laser stimulation were calculated.

PDC is a frequency domain representation of the key concept of *Granger *causality. Briefly, if knowledge of *x*(n)'s past significantly improves prediction of *y*(n), we could then states that an observed time-series *x*(n) *Granger*-causes series *y*(n). This relation between time-series is by no means reciprocal. Absence of PDC between two structures at a given frequency means the lack of a direct link between them. Thus, PDC allows the detection of coactivations among simultaneous neuronal activities by highlighting one neuronal group that possibly drives another. The detailed methodology of PDC has been described elsewhere [53-57]. In the present study, principal component analysis (PCA) for neurons in each brain area was first performed in *Nex*. Then the first principal component (PC1) of a given brain area that had the largest response were exported into *MatLab*, and the value of PDC across 1 – 50 Hz for each 2.5-s analysis time window were calculated. These values were then averaged around the laser stimulation events (0–10 s post-stimulus) and normalized to Z scores relative to the baseline (before stimulation) data.

ANOVAs and non-parametric *Chi*-square tests were performed to determine differences in *Z *scores and percentage of correlated neurons between sessions, respectively. *Bonferroni*'s test was used for *post hoc *test and *p *< 0.05 was considered to be level of significance for all the statistics.

## Abbreviations

ACC: Anterior cingulate cortex; MD: Medial dorsal thalamus; SI: primary somatosensory cortex; PAG: Periaqueductal grey; IC: Insular cortex; PFC: Prefrontal cortex; PSTH: Peri-event time histogram; PDC: Partial directed coherence; PCA: Principal component analysis.

## Competing interests

The authors declare that they have no competing interests.

## Authors' contributions

JYW participated in the design of the study and drafted the manuscript. HTZ carried out all the experiment and performed the statistical analysis. JYC and DJW assisted with the electrophysiological recordings and data analysis. LAB contributed to the data analysis and interpretation. FL conceived of the study, and participated in its design and helped to draft the manuscript. All authors read and approved the final manuscript.
